# Editorial: Cancer genetics and epigenetics: theranostic targets and mechanisms

**DOI:** 10.3389/fgene.2024.1446474

**Published:** 2024-07-26

**Authors:** Aisha Farhana, Nabiha Yusuf, Zafar Rasheed

**Affiliations:** ^1^ Department of Clinical Laboratory Sciences, College of Applied Medical Sciences, Jouf University, Sakaka, Aljouf Province, Saudi Arabia; ^2^ Department of Dermatology, Heersink School of Medicine, University of Alabama at Birmingham, Birmingham, AL, United States; ^3^ Department of Pathology, College of Medicine, Qassim University, Buraidah, Qassim, Saudi Arabia

**Keywords:** cancers, epigenetics, genetics, cancer theranostics, cancer mechanisms, tumor biomarkers, mutations

Cancer poses a threat to global health and is among the leading causes of mortality worldwide. Hence, a mechanistic understanding of cancer aimed toward developing successful interventions mandates particular focus. Being a multifactorial disease, the development and progress of cancer intercepts genetic alterations, epigenetic dysregulation, and environmental influences. These research topics highlight the growing knowledge of the genetic and epigenetic mechanisms of cancer, and how it can be harnessed for successful therapeutic interventions. Cancer initiates and progresses through genetic and epigenetic alterations fostered by environmental and genetic interactions (Ocaña-Paredes et al., 2024). Most cancer-causing mutations damage the DNA sequence, i.e., genetic mutations, while others are dynamic and heritable but independent of the DNA sequence, i.e., epigenetic mutations. DNA mutations may be irreversible point mutations, chromosomal rearrangement, deletion, duplication, etc., or reversible epigenetic changes like alterations in methylation patterns and histone posttranslational modifications. Epigenetic mutations can disrupt methylation patterns and modify histones and nucleosome positioning to alter gene expression. Also, inactivating genetic mutations within the epigenome alters the epigenomic machinery (Ushijima et al., 2021). Therefore, an interplay of genomic and epigenomic circuits is the basis of cancer development and progression.

Genetic mutations are hardwired resilient changes in the genome, while epigenetic modifications are softwired and more vulnerable to therapeutic intervention. The current hurdles in the diagnostic and therapeutic discoveries against cancers have necessitated a deeper mechanistic understanding of cancer at the genetic-epigenetic interface (Farhana et al., 2021). Interestingly, besides genetic underpinning, recent reports have also identified solely epigenetic mechanisms of cancer using *Drosophila* models. Transient disturbances in some genes can induce irreversible deregulation of cancer-associated genes, leading to tumorigenesis (Parreno et al., 2024). Furthermore, cancer-associated abnormalities mosaics have been identified in every chromatin pathway domain, spanning histones, histone effectors, chromatin remodeling machinery, transcription factors, DNA modifiers, etc. Genetic alteration of small and large magnitude perturb the healthy human epigenome and can serve as a malleable therapeutic target, thereby strengthening the impact of epigenetic modulations in driving various cancers (Gryder et al., 2022).

This Research Topic encompasses an explorative review of genetic mutation leading to methylthioadenosine phosphorylase (MTAP) deficiency as a target for cancer therapeutics by Fan et al. MTAP loss occurs as part of 9p21 loss and has an overall prevalence of 8% (Nilforoushan and Moatamed, 2020). Understanding the intricate and dynamic function of MTAP mutations in various cancers holds the therapeutic success of MTAP deleted tumors. The review details the molecular mechanisms and structural insights into MTAP and the potential of manipulating protein arginine methyltransferase 5 (PRMT5) and methionine adenosyltransferase 2A (MAT2A) as therapeutic targets in such tumors. Another outstanding work from this Research Topic by Awah et al., discovered that ERBB2^+^ cancers, which leads to many cancer deaths, harbors mRNA stabilizing poly U sequences on their 3′UTR. The researchers developed a novel method to synthesize unstable ERBB2 mRNA-stabilizing sequences. The engineered sequences were effective and competent against the endogenous ERBB2 mRNA-encoded sequences (Liu et al., 2021). This conclusively demonstrates the effect of sequence manipulation to degrade ERBB2 transcripts and subsequent protein loss in various cancer cell types in drug-resistant and wild-type cancers, *in vitro* and *in vivo*. The innovative approach developed by Awah et al. can be effectively expanded to other oncogenic signals to mitigate their effect, which lead to therapeutic failure.

The impact of ovarian cancer on global cancer-associated mortality is daunting due to prognostic and therapeutic challenges (Liu et al., 2021). Even though numerous research efforts are directed toward ovarian cancers, the identification of significant biomarkers remains far-fetched (Yousefi et al., 2020). Chen et al., comprehensively reviewed ferroptosis as a potent marker for the diagnosis, prognosis, and therapy for ovarian cancers (Li et al., 2020). Ferroptosis, a regulated cell death pathway distinct from apoptosis, necrosis, and autophagy, lies at the junction of various tumors’ initiation, progression, and metastatic phases. Their work provides a knowledge base of ferroptosis, covering its genetics, mechanistic understanding, signaling pathways, clinical features, and functional significance in ovarian cancers, highlighting the prospects of ferroptosis as a biomarker and a treatment modality for ovarian cancers (Chen and Liu, 2024; Ngoi et al., 2024).

Further, in a comprehensive systematic review and metanalysis, You et al. presented an overview of the association of methylenetetrahydrofolate reductase (MTHFR) gene polymorphisms, C677T and A1298C, for the developmental risk of prostate cancers. The systematic review of 26 case-control studies spanning control and cases for C677T and A1298C polymorphism did not demonstrate significant risk association with MTHFR genes. Nonetheless, they identified a lower cancer risk in the Asian population harboring C677T polymorphism and an increased risk among several races in the United States harboring A1298C polymorphism. Notably, the study seeks to resolve the contradicting research outcomes underpinning the association of prostate cancer risk with MTHFR gene polymorphism, identifying that C677T and A1298C polymorphisms influence on prostate cancer risk is specific to the populations.

Though the genetic and epigenetic knowledge of cancers is expansive, existing lacunae in identifying strategic genetic-epigenetic nodes are impeding the progress of the field toward advanced theranostic and socioeconomic goals. Overall, the studies in this Research Topic establish new knowledge about cancer genetics and epigenetics mechanisms, opening novel diagnostic and therapeutic fronts. The studies published in these Research Topic and other similar focused Research Topic prompt subsequent exploration of genetic and epigenetic biomarkers, mechanistic drivers of cancer, cancer classification, drug resistance mechanisms, etc. Identifying the intricate relationship between genetics and epigenetics and the modular node that skews average cellular growth and development towards cancerous phenotype can help precise diagnostic and therapeutic intervention for various cancers (Morel et al., 2020). Since cancer epigenomic orchestrates responses to therapeutic interventions, expanding our knowledge of the landscape of cancer genetic-epigenetics interface is imperative in the clinical course of precision medicine. The field has advanced to developing medications targeting epigenetic modulators, which have shown promise in treating solid tumors in preclinical and clinical trials (Kan et al., 2022). Cutting-edge epigenetic therapy offers unique insights into several cancer treatment models that identify aberrant epigenetic modifications (Davalos and Esteller, 2023). This is a significant advancement in treating malignant tumors and tailored precision diagnostics. Advancements in technology, such as artificial intelligence and sequencing techniques, along with the ongoing progress in epigenetic therapies, will create new opportunities for developing precision diagnostics and treatments.

Conclusion: The Research Topic presents significant knowledge about the genetic and epigenetic nexus of cancer development and subsequent translational tracks that can be derived from this knowledge. Nevertheless, further research is warranted to draw prognostic, diagnostic, and therapeutic courses toward targeted cancer modalities and precision medicine.
